# Smoking and risk of breast cancer in the Generations Study cohort

**DOI:** 10.1186/s13058-017-0908-4

**Published:** 2017-11-22

**Authors:** Michael E. Jones, Minouk J. Schoemaker, Lauren B. Wright, Alan Ashworth, Anthony J. Swerdlow

**Affiliations:** 1 Division of Genetics and Epidemiology, The Institute of Cancer Research, London, SW7 3RP UK; 2 Division of Breast Cancer Research, The Institute of Cancer Research, London, SW7 3RP UK; 3 Breakthrough Breast Cancer Research Centre, The Institute of Cancer Research, London, SW7 3RP UK; 4 Division of Molecular Pathology, The Institute of Cancer Research, London, SW7 3RP UK; 5 Present Address: UCSF Helen Diller Family Comprehensive Cancer Center, San Francisco, CA 94158 USA

**Keywords:** Smoking, Breast neoplasms, Cohort studies

## Abstract

**Background:**

Plausible biological reasons exist regarding why smoking could affect breast cancer risk, but epidemiological evidence is inconsistent.

**Methods:**

We used serial questionnaire information from the Generations Study cohort (United Kingdom) to estimate HRs for breast cancer in relation to smoking adjusted for potentially confounding factors, including alcohol intake.

**Results:**

Among 102,927 women recruited 2003–2013, with an average of 7.7 years of follow-up, 1815 developed invasive breast cancer. The HR (reference group was never smokers) was 1.14 (95% CI 1.03–1.25; *P* = 0.010) for ever smokers, 1.24 (95% CI 1.08–1.43; *P* = 0.002) for starting smoking at ages < 17 years, and 1.23 (1.07–1.41; *P* = 0.004) for starting smoking 1–4 years after menarche. Breast cancer risk was not statistically associated with interval from initiation of smoking to first birth (*P*-trend = 0.97). Women with a family history of breast cancer (ever smoker vs never smoker HR 1.35; 95% CI 1.12–1.62; *P* = 0.002) had a significantly larger HR in relation to ever smokers (*P* for interaction = 0.039) than women without (ever smoker vs never smoker HR 1.07; 95% CI 0.96–1.20; *P* = 0.22). The interaction was prominent for age at starting smoking (*P* = 0.003) and starting smoking relative to age at menarche (*P* = 0.0001).

**Conclusions:**

Smoking was associated with a modest but significantly increased risk of breast cancer, particularly among women who started smoking at adolescent or peri-menarcheal ages. The relative risk of breast cancer associated with smoking was greater for women with a family history of the disease.

**Electronic supplementary material:**

The online version of this article (doi:10.1186/s13058-017-0908-4) contains supplementary material, which is available to authorized users.

## Background

The carcinogenic potential of tobacco smoke is unarguable [1, 2], and there are plausible biological reasons why smoking could affect breast cancer risk [2–5]. Authors of reviews of the association between cigarette smoking and breast cancer up to 2004 did not, however, generally find conclusive evidence for a causal relationship in humans [5–7]. Authors of more recent epidemiological analyses have reported modest raised risks with current [8–19] or former [8–15, 20] smoking, but questions remain about the extent to which this association is a consequence of confounding by alcohol use, whether risk is increased if smoking starts in adolescence or before first childbirth, and whether risk is modified by family history of breast cancer [1, 2]. We therefore examined the risk of invasive breast cancer in relation to smoking in a large cohort study using detailed questionnaire information at recruitment and during follow-up, with adjustment for alcohol consumption and other potentially confounding factors.

## Methods

The Generations Study is a cohort study of over 113,700 women aged 16 years or older from the United Kingdom, from whom questionnaire information and informed consent were gained at recruitment since 2003 [21]. Initial recruits to the cohort were from women involved in the breast cancer charity that funded the study, as well as from women who responded to publicity about the study. Women who joined the study were asked to nominate female friends and family members, who were then contacted about joining the study. This referral method continued with subsequent recruits [21]. The first follow-up questionnaire (2½ years after recruitment) was completed by 99% of non-deceased participants, a second (6 years after recruitment) by 96%, and a third (9½ years after recruitment) by 94% (of those recruited long enough ago to have entered this round of follow-up). The study was approved by the South East Multi-Centre Research Ethics Committee.

Breast and other cancers occurring in the cohort were identified from recruitment and follow-up questionnaires, spontaneous reports to the study centre, and from ‘flagging’ (*see below*) for those lost to questionnaire follow-up. Confirmation of diagnosis was obtained from cancer registries in the United Kingdom, ‘flagging’ at the National Health Service Central Registers (virtually complete registers of the populations of England and Wales, and of Scotland, to which study participants can be linked and on which deaths, cancer registrations and emigrations are ‘flagged’ and then periodically reported to authorized medical researchers), pathology reports and correspondence with patients’ general practitioners.

Information on risk factors for breast cancer was obtained from recruitment and follow-up questionnaires. In relation to smoking, women were asked if they had ‘ever smoked regularly (i.e., most days for at least 6 months)’, if they still smoked regularly, age started and stopped, and number of cigarettes smoked per day in different periods of their lives (during ages 16–24, 25–49, 50+ years). For analysis, we defined the period of ‘current smoking’ to include both current smokers and the year immediately after stopping, to avoid potential ‘reverse-causation’ bias from women who may have stopped smoking during the workup to a formal breast cancer diagnosis. For alcohol use, we asked women if they had been a regular drinker ‘in the sense of drinking at least one glass of alcohol per week on average’, ages started and stopped, and quantity consumed in different periods of life (during ages 18–24, 25–49, 50+ years). We converted the quantity of alcohol consumed in each period of life into daily grams of alcohol. We split into three groups the women who reported current drinking (<60 g/day, ≥ 60 g/day, and amount unknown), and we classified women who had reported stopping drinking as former drinkers. For some women, we did not know their current drinking status during follow-up, but we knew they had consumed alcohol in the past, and these women were classified as ‘ever drinkers’. Because we had collected ages or dates at which certain events or changes in lifestyle occurred, we were able to update smoking status, alcohol use, parity, oral contraceptive (OC) use, menopausal hormone therapy (MHT) use and menopausal status at the ages these episodes occurred through the time of the second follow-up questionnaire. We updated duration of smoking for current smokers, as well as time since cessation for former smokers, in yearly increments, using smoking start and stop ages from the recruitment and second follow-up questionnaires. We updated cigarettes smoked per day, pack-years smoked, alcohol consumption and post-menopausal body mass index (BMI) at the date of the second follow-up questionnaire.

### Statistical analysis

The present analytic cohort is based on all women who were recruited into the study during June 2003–December 2013 without prior invasive or in situ breast cancer or other malignant cancer (except non-melanoma skin cancer) or prior mastectomy. The recruitment cut-off at December 2013 was selected because at the time of analysis the second follow-up was practically complete for this group of recruits, two-thirds of the cohort had reached the third follow-up, and we had ‘flagging’ information to June 2017. Women entered risk at their date of recruitment and were censored at the earliest date of invasive breast cancer or in situ breast cancer; other malignancy (except non-melanoma skin cancer); death; most recent follow-up questionnaire (depending on date of recruitment) if completed, or the date the most recent follow-up questionnaire was due if cancer and vital status was known from ‘flagging’; or previously completed questionnaire if lost to follow-up. We censored follow-up at in situ breast cancer or other malignancy because we reasoned that if smoking is related to risk of in situ breast cancer or other malignancy, and ensuing treatments or their consequences alter risk of subsequent invasive breast cancer, including subsequent follow-up may obscure associations between smoking and invasive breast cancer.

Left-truncated and right-censored Cox proportional hazards regression [22] with attained age as the implicit time scale was used to estimate HR and 95% CI for smoking and risk of first invasive breast cancer. We adjusted for time since recruitment to cohort (0, 1–2, 3+ years); birth cohort (1908–1939, 1940–1949, 1950–1959, 1960–1969, 1970–1996); benign breast disease (yes/no); family history of breast cancer in first-degree relatives (yes/no); socio-economic score (Acorn score (https://acorn.caci.co.uk/) as trend, or missing indicator); age at menarche (trend, or missing indicator); age at first pregnancy (trend, or missing indicator); parity (trend, or missing indicator); duration of breastfeeding (trend, or missing indicator); current OC use during follow-up, before menopause (yes/no); alcohol consumption (trend for current drinker 1 to < 60 g/day, indicator variables for never regular, current drinker ≥ 60 g/day, past drinker, drinker with unknown details); physical activity [log(metabolic equivalent) trend, missing indicator]; pre-menopausal BMI at age 20 years (trend, or missing indicator); post-menopausal BMI (trend, or missing indicator); MHT use (never used, ex-user, current oestrogen-only user, current oestrogen plus progestogen user, current user of other types, missing indicator); menopausal status (pre- or post-menopausal); and age at menopause (trend, or missing indicator). BMI was used to create two separate variables: pre-menopausal BMI (potentially available for all women) and post-menopausal BMI (only available at post-menopausal ages). We used BMI at age 20 to represent pre-menopausal BMI. Separately, if a woman was post-menopausal at entry to the cohort we used her BMI at entry for her post-menopausal BMI (and if she was pre-menopausal at this time, post-menopausal BMI was unknown). If a woman was post-menopausal at the time of the follow-up questionnaire, we updated from this point in time her post-menopausal BMI with the value from this follow-up questionnaire. Statistical trends were evaluated using continuous values, except for duration and time since cessation of smoking, which were based on discrete time-varying, annually updated values. For trend analyses where there was an unexposed group (e.g., never smokers in analyses of smoking duration), the unexposed group was not assigned a zero magnitude but was treated as a separate categorical term, as was any missing value group. In particular we adjusted our analyses of smoking and breast cancer for alcohol using daily current alcohol consumption as a continuous measure if within the range 1 to < 60 g/day, and categorical terms for non-drinkers, for those with consumption ≥ 60 g/day (because we did not want a minority of women who reported very high consumption to influence unduly the trend with daily consumption), past drinkers, and those for whom details of consumption were missing, by fitting appropriate interaction terms in the Cox regression model. Heterogeneity in HRs by subtype of breast cancer defined by oestrogen receptor (ER) status or morphology was assessed using a data augmentation method [23] and Wald chi-square tests [24]. All statistical tests were two-sided, and analyses were conducted using Stata/IC version 14.0 software [25].

## Results

During 2003–2013, a recruitment questionnaire was completed by 102,940 women who had no previous invasive or in situ breast cancer or other malignancy (except non-melanoma skin cancer). At censoring date, 1.1% of women had died. Of the remainder, cancer and vital status were known for 96.5% who had completed the relevant follow-up questionnaire and for a further 2.4% from ‘flagging’ at the National Health Service Central Registers. The remaining 1.1% were lost to follow-up at an earlier date. Thirteen women (including one with breast cancer) were excluded from subsequent analyses because of self-contradictory information for parity or smoking, leaving 102,927 subjects for analysis.

Table 1 presents descriptive characteristics at recruitment of the cohort eligible for analysis. The median age at recruitment was 47 years (IQR 36–57). A majority of participants (64.1%) reported never smoking, but only 10.3% were never-regular consumers of alcohol. In relation to alcohol consumption, 12.5% of never smokers were non-drinkers, in contrast to 6.4% of ever smokers. Among those who reported drinking < 60 g/day, the median alcohol consumption (g/day) was 14.2 (IQR 8.7–22.1) among never smokers and 19.0 (IQR 11.9–29.2) among ever smokers. Additional file 1: Table S1 provides further descriptive characteristics of the cohort in relation to age at starting smoking, thelarche, parity, menopausal status and BMI.

**Table 1:** Characteristics of 102,927 women from the Generations Study, recruited 2003–2013

Study population	No. of subjects	%
Year of birth		
1908–1939	5917	5.8
1940–1949	21,488	20.9
1950–1959	24,735	24.0
1960–1969	23,749	23.1
1970–1996	27,038	26.3
Year of recruitment		
2003–2005	33,807	32.9
2006–2007	48,489	47.1
2008–2013	20,631	20.0
Age at recruitment, years		
16–34	23,289	22.6
35–44	23,116	22.5
45–54	24,385	23.7
55–64	23,837	23.2
65–74	7308	7.1
75–102	992	1.0
Age at menarche, years		
7–11	20,658	20.1
12–14	62,291	60.5
15–19	9353	9.1
Not known or anomalous^a^	10,625	10.3
Family history of breast cancer in first-degree relatives		
No	87,030	84.6
Yes	15,897	15.4
Smoking status, at entry into cohort		
Never smoked	66,013	64.1
Current smoker	8491	8.3
Former smoker	28,300	27.5
Ever smoker, status unknown at this time	123	0.1
Alcohol consumption, at entry into cohort		
Never regular	10,574	10.3
Current drinker (< 60 g/day)	63,690	61.9
Current drinker (≥ 60 g/day)	1280	1.2
Current drinker, amount unknown	4306	4.2
Former drinker	13,513	13.1
Ever drinker, status unknown at this time	9564	9.3
Total number of subjects	102,927	100.0

Total number of subjects excludes 13 women (including 1 who developed breast cancer) with self-contradictory information for parity or smoking

^a^Includes 2 women reporting menarche before age 5 years, 23 at ages 20–33 years, and 23 reporting never having periods

During 788,361 person-years (median 6.6 years; mean 7.7 years) of follow-up, 1815 invasive breast cancers were diagnosed, of which 1813 were confirmed through national cancer registration or medical records, and the remaining 2 were self-reported with treatments that imply breast cancer. ER status data were available for 99.3%, and of these 83.7% were ER-positive. Invasive ductal carcinoma accounted for 78.8%, and lobular 16.4%, of tumours. Further descriptive characteristics of the breast cancer cases are given in Additional file 1: Table S2.

The HR for invasive breast cancer in relation to ever smoking was 1.17 (95% CI 1.07–1.29; *P* = 0.0009) when adjusted only for attained age, 1.13 (95% CI 1.03–1.24; *P* = 0.012) when also adjusted for alcohol consumption, and 1.14 (95% CI 1.03–1.25; *P* = 0.010) when further adjusted for other potentially confounding variables (*see* the Methods section above and Table 2). All subsequent results are adjusted for attained age, alcohol consumption and the potentially confounding variables, unless otherwise stated.

**Table 2:** Relative risk of breast cancer in relation to smoking status, intensity, duration, dose, and cessation, by oestrogen receptor status

	Oestrogen receptor status								All breast cancer^a^			
	Positive				Negative							
	Cases	HR^b^	95% CI	*P* value	Cases	HR^b^	95% CI	*P* value	Cases	HR^b^	95% CI	*P* value
Ever previously smoked cigarettes^c^												
Never	898	1.00	Baseline		167	1.00	Baseline		1073	1.00	Baseline	
Ever	611	1.12	1.01–1.24	0.035	127	1.25	0.99–1.58	0.063	742	1.14	1.03–1.25	0.010
	Ever smoking by oestrogen receptor status interaction *P* = 0.40											
Cigarettes per day, averaged over years when smoking^d^												
Never smoked	898	1.00	Baseline		167	1.00	Baseline		1073	1.00	Baseline	
1–4	216	1.07	0.92–1.25	0.36	41	1.11	0.79–1.56	0.54	258	1.08	0.94–1.24	0.29
5–9	117	1.27	1.05–1.55	0.016	17	0.97	0.59–1.59	0.89	135	1.22	1.02–1.47	0.031
10–14	68	1.37	1.07–1.76	0.013	13	1.34	0.76–2.36	0.31	82	1.37	1.09–1.72	0.0070
15+	51	1.48	1.11–1.97	0.0079	11	1.61	0.87–2.97	0.13	62	1.48	1.14–1.92	0.0031
Amount unknown	159	0.94	0.79–1.12	0.51	45	1.46	1.05–2.03	0.025	205	1.03	0.88–1.20	0.71
	Trend^e^ *P* = 0.023				Trend^e^ *P* = 0.11				Trend^e^ *P* = 0.0060			
	Trend interaction *P* = 0.52											
Duration of smoking, years^f^												
Never smoked	898	1.00	Baseline		167	1.00	Baseline		1073	1.00	Baseline	
1–9	139	0.95	0.79–1.13	0.57	37	1.30	0.91–1.85	0.15	177	1.00	0.85–1.18	0.97
10–19	187	1.22	1.04–1.43	0.015	38	1.26	0.88–1.79	0.21	225	1.21	1.05–1.41	0.009
20–29	113	1.16	0.95–1.41	0.15	28	1.59	1.06–2.38	0.025	141	1.21	1.02–1.45	0.033
30+	137	1.23	1.03–1.48	0.024	20	1.07	0.67–1.71	0.78	159	1.22	1.02–1.44	0.026
Duration unknown	35	0.97	0.69–1.36	0.86	4	0.61	0.23–1.66	0.34	40	0.93	0.68–1.28	0.66
	Trend^e^ *P* = 0.17				Trend^e^ *P* = 0.85				Trend^e^ *P* = 0.24			
	Trend interaction *P* = 0.44											
Pack-years of smoking^d^												
Never smoked	898	1.00	Baseline		167	1.00	Baseline		1073	1.00	Baseline	
1 to < 5	156	1.13	0.95–1.34	0.15	25	0.93	0.61–1.42	0.75	182	1.10	0.94–1.29	0.25
5 to < 10	84	1.03	0.82–1.29	0.81	19	1.21	0.75–1.94	0.44	103	1.05	0.85–1.28	0.66
10 to < 20	114	1.25	1.03–1.53	0.027	24	1.42	0.92–2.18	0.11	138	1.27	1.06–1.52	0.010
20+	98	1.48	1.19–1.83	0.0003	14	1.22	0.71–2.13	0.47	114	1.45	1.19–1.77	0.0002
Amount unknown	159	0.95	0.80–1.13	0.59	45	1.47	1.06–2.05	0.022	205	1.03	0.88–1.20	0.70
	Trend^e^ *P* = 0.024				Trend^e^ *P* = 0.16				Trend^e^ *P* = 0.0069			
	Trend interaction *P* = 0.66											
Current or former smoking^c^												
Never smoked	898	1.00	Baseline		167	1.00	Baseline		1073	1.00	Baseline	
Current^g^	73	1.14	0.89–1.45	0.30	14	1.07	0.62–1.86	0.80	87	1.12	0.89–1.39	0.34
Former	538	1.12	1.00–1.25	0.047	113	1.27	1.00–1.62	0.50	655	1.14	1.03–1.26	0.011
	Smoking status by oestrogen receptor status interaction *P* = 0.58											
Time since cessation, years^f^												
Never smoked	898	1.00	Baseline		167	1.00	Baseline		1073	1.00	Baseline	
Current^g^ smoker	73	1.14	0.90–1.46	0.28	14	1.08	0.62–1.87	0.79	87	1.12	0.90–1.40	0.31
1–9	100	1.25	1.01–1.54	0.040	26	1.48	0.98–2.23	0.064	127	1.28	1.06–1.55	0.009
10–19	115	1.17	0.96–1.42	0.12	28	1.42	0.94–2.13	0.10	145	1.21	1.02–1.45	0.032
20–29	133	1.10	0.91–1.32	0.33	20	0.94	0.59–1.51	0.81	153	1.07	0.90–1.27	0.46
30+	170	1.05	0.88–1.24	0.61	38	1.43	0.99–2.06	0.058	209	1.10	0.94–1.28	0.24
Duration unknown	20	1.06	0.68–1.65	0.81	1	0.29	0.04–2.08	0.22	21	0.93	0.60–1.43	0.74
	Trend^e^ *P* = 0.055				Trend^e^ *P* = 0.85				Trend^e^ *P* = 0.071			
	Trend interaction *P* = 0.55											

^a^Includes 12 breast cancers with unknown or unmeasured oestrogen receptor status

^b^Adjusted for attained age (Cox regression time scale); time since recruitment to cohort (0, 1–2, 3+ years); birth cohort (1908–1939, 1940–1949, 1950–1959, 1960–1969, 1970–1996); benign breast disease (yes/no); family history of breast cancer in first-degree relatives (yes/no); socio-economic score (Acorn score as trend, missing); age at menarche (trend, missing); age at first pregnancy (trend, missing); parity (trend, missing); duration of breastfeeding (trend, missing); current oral contraceptive use before menopause (yes/no); alcohol consumption (never regular, trend current drinker 1 to < 60 g/day, current drinker ≥ 60 g/day, past drinker, drinker with unknown details); physical activity [log(metabolic equivalent) trend, missing]; pre-menopausal body mass index at age 20 years (trend, missing); post-menopausal body mass index (trend, missing); menopausal hormone therapy use (never used, ex-user, current oestrogen-only user, current oestrogen plus progestogen user, current user of other types, missing); menopausal status (pre- or post-menopausal) and age at menopause (trend, missing)

^c^Time updated through follow-up period

^d^Time updated at the point renewed information was available from follow-up questionnaire

^e^Trend excludes never smoker and unknown group

^f^Time updated in yearly steps

^g^Includes current smokers and < 1 year immediately after stopping

Table 2 presents results for breast cancer overall and by ER status. The HR for ever smoking was raised for ER-positive (HR 1.12; 95% CI 1.01–1.24; *P* = 0.035) and ER-negative (HR 1.25; 95% CI 0.99–1.58; *P* = 0.063) breast cancer, and the difference between the HRs was not significant (*P* = 0.40). Breast cancer risk increased significantly with number of cigarettes smoked per day for all breast cancer (*P*-trend = 0.0060) and for ER-positive tumours (*P*-trend = 0.023). Breast cancer risks were raised significantly after 10+ years’ duration of smoking (10+ years vs never smoking *P* = 0.0004). Breast cancer risks did not further rise beyond 10 years’ duration, and because of this non-linear relationship, there was no significant linear trend with duration of smoking (*P*-trend = 0.24), nor was there significant heterogeneity in the trend by ER status. Pack-years of smoking was associated with breast cancer risk overall (*P*-trend = 0.0069) and ER-positive breast cancer (*P*-trend = 0.024) but not for ER-negative (*P*-trend = 0.16) tumours; there was no significant heterogeneity of the pack-years trend by ER status (*P* = 0.66).

The HR within the year after smoking cessation was 2.68 (95% CI 1.60–4.46), based on 15 cases, but for reasons described in the Methods section above, this risk period was assigned for further analysis to the ‘current smoker’ group. On this basis, risk of breast cancer was raised in current (HR 1.12; 95% CI 0.89–1.39; *P* = 0.34) and former (HR 1.14; 95% CI 1.03–1.26; *P* = 0.011) smokers, although only the latter reached statistical significance; there was no significant heterogeneity by ER status. Breast cancer risks were significantly raised within the first 20 years after cessation of smoking and decreased with greater time since cessation, although the trend was not significant (*P*-trend = 0.071), and there was no significant heterogeneity in this trend by ER status.

There was significant variation in risk of breast cancer by age at start of smoking (Table 3) (*P*-heterogeneity = 0.018; not presented in Table 3). Breast cancer risk was significantly increased if smoking started at age < 17 years (HR 1.24; 95% CI 1.08–1.43; *P* = 0.0023) or 17–19 years (HR 1.15; 95% CI 1.01–1.31; *P* = 0.030) relative to non-smokers, but not if it started at older ages. The risk was significantly increased for ER-positive subjects, only for smokers starting at ages < 17 years, and no significant risk increase was noted for ER-negative breast cancer. When adjusted for pack-years, the breast cancer risk (HR) for starting smoking at age < 17 years was 1.12 (95% CI 0.96–1.32; *P* = 0.14), and when adjusted for duration of smoking, it was 1.16 (95% CI 0.96–1.40; *P* = 0.11) (not presented in Table 3).

**Table 3:** Relative risk of breast cancer in relation to smoking in relation to age at start of smoking, by oestrogen receptor status

	Oestrogen receptor status								All breast cancer^a^			
	Positive				Negative							
	Cases	HR^b^	95% CI	*P* value	Cases	HR^b^	95% CI	*P* value	Cases	HR^b^	95% CI	*P* value
Age started smoking, years^c^												
Never smoked	898	1.00	Baseline		167	1.00	Baseline		1073	1.00	Baseline	
< 17	218	1.25	1.08–1.46	0.0032	42	1.22	0.87–1.72	0.25	261	1.24	1.08–1.43	0.0023
17–19	249	1.13	0.98–1.30	0.093	54	1.32	0.97–1.80	0.077	304	1.15	1.01–1.31	0.030
20+	122	0.95	0.79–1.15	0.62	28	1.25	0.84–1.87	0.28	151	1.00	0.84–1.18	0.96
Age unknown	22	0.94	0.62–1.44	0.79	3	0.73	0.23–2.30	0.59	26	0.94	0.64–1.39	0.76
	Trend^d^ *P* = 0.17				Trend^d^ *P* = 0.74				Trend among ever smokers^d^ *P* = 0.18			
	Trend interaction *P* = 0.30											
Cigarettes per day at ages 16–24 years (among those aged ≥ 25 years at entry)												
Never smoked	895	1.00	Baseline		166	1.00	Baseline		1069	1.00	Baseline	
1–4	77	1.10	0.87–1.39	0.42	21	1.65	1.05–2.60	0.032	99	1.19	0.97–1.46	0.10
5–9	131	1.23	1.02–1.48	0.027	18	0.92	0.57–1.50	0.75	149	1.16	0.99–1.40	0.069
10–14	170	1.11	0.94–1.31	0.22	31	1.07	0.73–1.57	0.73	203	1.10	0.95–1.29	0.21
15+	169	1.13	0.96–1.34	0.14	42	1.44	1.02–2.03	0.038	212	1.18	1.01–1.37	0.034
Started at age 25+	31	0.91	0.64–1.31	0.62	7	1.22	0.57–2.60	0.61	38	0.95	0.69–1.31	0.75
Unknown	31	0.97	0.68–1.39	0.87	7	1.32	0.62–2.79	0.47	38	1.01	0.73–1.40	0.95
	Trend^d^ *P* = 0.70				Trend^d^ *P* = 0.65				Trend^d^ *P* = 0.78			
	Trend interaction *P* = 0.81											
Starting smoking relative to age at menarche, years												
Never smoked	898	1.00	Baseline		167	1.00	Baseline		1073	1.00	Baseline	
At or before menarche	32	1.60	1.12–2.28	0.010	2	0.50	0.12–2.02	0.33	34	1.40	0.98–1.99	0.061
Years started after menarche												
1–4	217	1.21	1.04–1.41	0.015	48	1.38	1.00–1.91	0.049	266	1.23	1.07–1.41	0.0040
5–9	239	1.09	0.94–1.27	0.23	54	1.35	0.99–1.84	0.060	295	1.13	0.99–1.29	0.071
10–14	36	1.03	0.74–1.44	0.86	7	1.13	0.53–2.43	0.75	43	1.04	0.76–1.41	0.82
15+	12	0.64	0.36–1.13	0.12	6	1.92	0.85–4.34	0.12	18	0.81	0.51–1.30	0.39
Interval unknown	75	1.02	0.79–1.32	0.88	10	0.74	0.38–1.41	0.35	86	0.99	0.77–1.27	0.92
	Trend^d^ *P* = 0.041				Trend^d^ *P* = 0.80				Trend^d^ *P* = 0.031			
	Trend for interaction *P* = 0.42											
Starting smoking relative to age at thelarche, years												
Never smoked	898	1.00	Baseline		167	1.00	Baseline		1073	1.00	Baseline	
At or before thelarche	16	1.45	0.88–2.39	0.14	1	0.45	0.06–3.24	0.43	17	1.27	0.79–2.06	0.33
Years started after thelarche												
1–4	160	1.17	0.99–1.39	0.075	31	1.17	0.79–1.72	0.43	192	1.17	1.00–1.37	0.056
5–9	238	1.06	0.92–1.22	0.47	60	1.42	1.06–1.91	0.020	300	1.11	0.98–1.27	0.10
10–14	37	0.98	0.70–1.36	0.90	11	1.60	0.87–2.95	0.13	48	1.07	0.80–1.43	0.66
15+	18	0.94	0.59–1.50	0.79	6	1.83	0.81–4.14	0.14	24	1.06	0.71–1.59	0.77
Interval unknown	142	1.22	1.02–1.46	0.031	18	0.87	0.53–1.42	0.59	161	1.17	0.99–1.38	0.074
	Trend^d^ *P* = 0.58				Trend^d^ *P* = 0.35				Trend among ever smokers^d^ *P* = 0.82			
	Trend for interaction *P* = 0.28											
Starting smoking relative to first childbirth, among parous (follow-up time only from first childbirth)												
Never smoked	772	1.00	Baseline		148	1.00	Baseline		927	1.00	Baseline	
After first childbirth	34	1.20	0.85–1.71	0.30	9	1.85	0.94–3.67	0.076	43	1.28	0.94–1.76	0.12
Interval from starting smoking to first childbirth, years												
1–4	73	1.12	0.88–1.44	0.35	15	1.28	0.74–2.21	0.38	89	1.14	0.91–1.45	0.23
5–9	177	1.06	0.90–1.25	0.51	42	1.35	0.96–1.90	0.089	220	1.10	0.94–1.28	0.22
10–14	130	1.08	0.89–1.30	0.42	22	0.89	0.57–1.40	0.62	152	1.04	0.87–1.24	0.65
15+	77	1.17	0.91–1.51	0.21	16	1.13	0.66–1.92	0.65	94	1.17	0.93–1.47	0.19
Interval unknown	22	1.07	0.70–1.64	0.75	2	0.54	0.13–2.17	0.38	25	1.02	0.69–1.52	0.92
	Trend^d^ *P* = 0.72				Trend^d^ *P* = 0.33				Trend among ever smokers^d^ *P* = 0.97			
	Trend for interaction *P* = 0.27											

^a^Includes 21 breast cancers with unknown or unmeasured oestrogen receptor status

^b^Adjusted for attained age (Cox regression time scale); time since recruitment to cohort (0, 1–2, 3+ years); birth cohort (1908–1939, 1940–1949, 1950–1959, 1960–1969, 1970–1996); benign breast disease (yes/no); family history of breast cancer in first-degree relatives (yes/no); socio-economic score (Acorn score as trend, missing); age at menarche (trend, missing); age at first pregnancy (trend, missing); parity (trend, missing); duration of breastfeeding (trend, missing); current oral contraceptive use before menopause (yes/no); alcohol consumption (never regular, trend current drinker 1 to < 60 g/day, current drinker ≥ 60 g/day, past drinker, drinker with unknown details); physical activity [log(metabolic equivalent) trend, missing]; pre-menopausal body mass index at age 20 years (trend, missing); post-menopausal body mass index (trend, missing); menopausal hormone therapy use (never used, ex-user, current oestrogen-only user, current oestrogen plus progestogen user, current user of other types, missing); menopausal status (pre- or post-menopausal) and age at menopause (trend, missing)

^c^Time updated through follow-up period

^d^Trend excludes never smoker and unknown group

In our questionnaire, we asked women only about the amount they smoked per day beginning at age 16 years; therefore, we could not examine smoking intensity at younger ages. There was no significant trend in breast cancer risk, however, in relation to cigarettes smoked per day at ages 16–24 years. Relative to age at menarche, breast cancer risks were highest if smoking started at or before menarche (HR 1.40; 95% CI 0.98–1.99; *P* = 0.061) or 1–4 years after (HR 1.23; 95% CI 1.07–1.41; *P* = 0.0040), with a significant downward trend in breast cancer risk with increasing interval from age at menarche to age at starting smoking (*P* = 0.031). A similar pattern was seen for ER-positive, but was less clear for ER-negative, breast cancer. A weaker relationship was seen with age at thelarche (e.g., 1–4 years after thelarche; HR 1.17; 95% CI 1.00–1.37; *P* = 0.056). When adjusted for pack-years of smoking, the HRs for age at start of smoking 1–4 years after menarche (HR 1.12; 95% CI 0.96–1.31; *P* = 0.15) or thelarche (HR 1.05; 95% CI 0.88–1.25; *P* = 0.59) were attenuated (not presented in Table 3). There was a comparable attenuation after adjusting for duration of smoking. Among parous women, there was a significant trend in breast cancer risk with interval from starting smoking to birth of first child (*P*-trend = 0.013); for an interval of 15+ years, the HR was 1.46 (95% CI 1.18–1.81; *P* = 0.0005). However, these results were not adjusted for age at first childbirth and parity (not presented in the tables), and when we adjusted (as shown in Table 3), there were no significantly raised HRs or trends for all breast cancer or by ER status.

When we analysed data by morphological type (Additional file 1: Table S3), we found significant associations for ductal breast cancer similar to the results for breast cancer overall, as well as generally non-significant results for lobular breast cancer. There were no significant interactions by morphological type in the risk of breast cancer with smoking.

There was no raised risk of breast cancer with ever smoking in non-drinkers (HR 0.97; 95% CI 0.61–1.52; *P* = 0.89) but a significantly raised breast cancer risk in those who had ever been drinkers (HR 1.18; 95% CI 1.07–1.30; *P* = 0.0010), although the difference in HRs was not significant (*P*-interaction = 0.41) (Table 4). When further stratified by amount of alcohol consumed, the HRs for ever smoking among current drinkers remained raised. The results were similar when we examined breast cancer risk by drinking status for former smokers relative to never smokers (Additional file 1: Table S4).

**Table 4:** Relative risk of breast cancer in relation to ever smoking, by level of alcohol consumption

Strata	Total number of cases in strata	HR for ever smoking relative to never smoking within strata of alcohol consumption^a^	95% CI	*P* value
Alcohol^b^				
Non-drinker	110	0.97	0.61–1.52	0.89
Ever drinker	1705	1.18	1.07–1.30	0.0010
		Interaction *P* ^c^ = 0.41		
Alcohol^b^				
Non-drinker	110	0.97	0.61–1.52	0.89
< 20 g/day	706	1.17	1.00–1.36	0.049
20 to < 40 g/day	356	1.17	0.95–1.44	0.13
40 to < 60 g/day	87	1.12	0.73–1.72	0.61
≥ 60 g/day	27	1.92	0.78–4.76	0.16
Current, amount unknown	180	1.47	1.09–1.98	0.011
Former drinker	295	0.98	0.78–1.25	0.89
Drinker, details missing	54	0.73	0.38–1.42	0.35
		Interaction *P* ^c^ = 0.33		

^a^Adjusted for attained age (Cox regression time scale); time since recruitment to cohort (0, 1–2, 3+ years); birth cohort (1908–1939, 1940–1949, 1950–1959, 1960–1969, 1970–1996); benign breast disease (yes/no); family history of breast cancer in first-degree relatives (yes/no); socio-economic score (Acorn score as trend, missing); age at menarche (trend, missing); age at first pregnancy (trend, missing); parity (trend, missing); duration of breastfeeding (trend, missing); current oral contraceptive use before menopause (yes/no); physical activity [log(metabolic equivalent) trend, missing]; pre-menopausal body mass index at age 20 years (trend, missing); post-menopausal body mass index (trend, missing); menopausal hormone therapy use (never used, ex-user, current oestrogen-only user, current oestrogen plus progestogen user, current user of other types, missing); menopausal status (pre- or post-menopausal) and age at menopause (trend, missing)

^b^Time updated through follow-up period

^c^Interaction across all categories, including missing groups

We examined further potential risk factor interactions with smoking but found no significant interactions with parity (*P* = 0.095), although for nulliparous ever smoking women, there was a statistically significantly increased risk of breast cancer (*P* = 0.012) (Additional file 1: Table S5), or menopausal status (*P* = 0.73) (Additional file 1: Table S6). However, although the HR of pre-menopausal ever smokers was somewhat larger than for post-menopausal ever smokers, the former did not reach statistical significance (*P* = 0.088), whereas the latter did (*P* = 0.040). Nor did we find significant interactions with birth cohort (*P* = 0.092), BMI at age 20 years (*P* = 0.55) or post-menopausal ages (*P* = 0.26), but we did see a significant interaction with family history of breast cancer (*P* = 0.038). There were significant interactions between family history and age at start of smoking (*P* = 0.0029) and starting smoking relative to age at menarche (*P* = 0.0001) in relation to risk of breast cancer (Table 5). In particular, among women with a family history of breast cancer, HRs were raised if smoking started at age 20+ years (HR 1.56; 95% CI 1.17–2.10; *P* = 0.0028) or < 20 years (HR 1.26; 95% CI 1.02–1.56: *P* = 0.029), and if started 5+ years after menarche (HR 1.53; 95% CI 1.22–1.91; *P* = 0.0002), and we noted that these were somewhat different from the results among women without a family history of breast cancer.

**Table 5:** Relative risk of breast cancer in relation to smoking, by family history of breast cancer

	Family history of breast cancer^a^							
	No				Yes			
	Cases	HR^b^	95% CI	*P* value	Cases	HR^b^	95% CI	*P* value
Ever previously smoked cigarettes^c^								
Never	819	1.00	Baseline		239	1.00	Baseline	
Ever	554	1.07	0.96–1.20	0.22	203	1.35	1.12–1.62	0.0018
	Interaction *P* = 0.038							
Age at start of smoking, years^c^								
Never smoker	819	1.00	Baseline		254	1.00	Baseline	
< 20	427	1.17	1.04–1.32	0.0098	138	1.26	1.02–1.56	0.029
20+	96	0.82	0.67–1.02	0.076	55	1.56	1.17–2.10	0.0028
Age unknown	16	0.75	0.46–1.23	0.26	10	1.57	0.84–2.96	0.16
	Interaction *P* = 0.0029							
		Trend^d^ *P* = 0.034				Trend^d^ *P* = 0.38		
	Trend interaction *P* = 0.044							
Starting smoking relative to age at menarche, years								
Never smoker	819	1.00	Baseline		254	1.00	Baseline	
Before or < 5 years	239	1.30	1.12–1.51	0.0006	61	1.06	0.80–1.41	0.68
5+ years	244	0.97	0.84–1.13	0.70	112	1.53	1.22–1.91	0.0002
Interval unknown	56	0.83	0.62–1.12	0.22	30	1.51	1.02–2.24	0.041
	Interaction *P* = 0.0001							
		Trend^d^ *P* = 0.0021				Trend^d^ *P* = 0.40		
	Trend interaction *P* = 0.0079							
Cigarettes per day age among ever smokers, averaged over years when smoking^e^								
Never smoker	819	1.00	Baseline		254	1.00	Baseline	
1–4	185	1.01	0.86–1.18	0.95	73	1.32	1.01–1.71	0.040
5+	210	1.28	1.09–1.49	0.0019	69	1.42	1.09–1.86	0.010
Unknown	144	0.94	0.79–1.13	0.51	61	1.31	0.99–1.74	0.059
	Interaction *P* = 0.13							
		Trend^d^ *P* = 0.0019				Trend^d^ *P* = 0.81		
	Trend interaction *P* = 0.18							
Duration of smoking among ever smokers, years^f^								
Never smoker	819	1.00	Baseline		254	1.00	Baseline	
1–9	129	0.94	0.78–1.14	0.54	48	1.20	0.88–1.64	0.24
10+	385	1.16	1.03–1.32	0.019	140	1.39	1.13–1.71	0.0020
Duration unknown	25	0.76	0.51–1.13	0.17	15	1.51	0.90–2.55	0.12
	Interaction *P* = 0.094							
		Trend^d^ *P* = 0.15				Trend^d^ *P* = 0.86		
	Trend interaction *P* = 0.37							
Pack-years of smoking among ever smokers^e^								
Never smoker	819	1.00	Baseline		254	1.00	Baseline	
1 to < 10	208	1.02	0.87–1.18	0.84	77	1.28	0.99–1.65	0.060
10+	187	1.30	1.11–1.53	0.0015	65	1.48	1.13–1.95	0.0051
Unknown	144	0.93	0.78–1.12	0.46	61	1.30	0.98–1.72	0.069
	Interaction *P* = 0.16							
		Trend^d^ *P* = 0.055				Trend^d^ *P* = 0.54		
	Trend interaction *P* = 0.37							

^a^First-degree relatives, at recruitment

^b^Adjusted for attained age (Cox regression time scale); time since recruitment to cohort (0, 1–2, 3+ years); birth cohort (1908–1939, 1940–1949, 1950–1959, 1960–1969, 1970–1996); benign breast disease (yes/no); socio-economic score (Acorn score as trend, missing); age at menarche (trend, missing); age at first pregnancy (trend, missing); parity (trend, missing); duration of breastfeeding (trend, missing); current oral contraceptive use before menopause (yes/no); alcohol consumption (never regular, trend current drinker 1 to < 60 g/day, current drinker ≥ 60 g/day, past drinker, drinker with unknown details); physical activity [log(metabolic equivalent) trend, missing]; pre-menopausal body mass index at age 20 years (trend, missing); post-menopausal body mass index (trend, missing); menopausal hormone therapy use (never used, ex-user, current oestrogen-only user, current oestrogen plus progestogen user, current user of other types, missing); menopausal status (pre- or post-menopausal) and age at menopause (trend, missing)

^c^Time updated through follow-up

^d^Trend excludes never smoker and unknown groups

^e^Time updated at the point renewed information was available from follow-up questionnaire

^f^Time updated in yearly steps

## Discussion

In the Generations Study cohort, we found a significant but modestly raised risk of invasive breast cancer in ever and former smokers, in women who smoked more than five cigarettes per day, had 10+ pack-years of use, or had stopped for < 20 years. Researchers in some previous studies have reported similar associations with smoking [8–17, 20], cigarettes per day [9–11, 19], pack-years [9–13, 17–19, 26–29], and cessation [8, 12, 19, 26, 28], but not all studies have shown these associations [10, 11, 13, 15–17, 19, 20, 29, 30]. We saw significantly raised risk with 10+ years’ duration of smoking, but no increasing trend beyond 10+ years. Increased risks at long durations (or significant trends) have previously been reported in some studies [8–13, 18–20, 26–28], although some classified non-smokers as smokers with zero duration [12, 20, 26, 28], and this may artefactually produce a significant trend which partly or wholly reflects the difference in risk between non-smokers and smokers (but this may not be the only reason for an association with 20+ years [long duration] of smoking).

We found that risk was significantly raised in former smokers, as previously reported [8–15, 20]. Risk was also raised with current smoking, but the number of current smokers in our cohort was small, and this result did not reach statistical significance, although researchers in some other studies have reported significantly raised risks in this group [8–19]. The raised risks for current and former smokers were similar (HR 1.12 and 1.14, respectively), and the CIs overlapped, suggesting, within our cohort, no material difference between current and former smokers in relation to breast cancer risk.

### Breast cancer subtypes

We found significant raised risks for ER-positive and ductal breast cancer, which were the most common types in our study, but no significant heterogeneity by ER status or morphological type of the breast cancer in relation to smoking. The statistical power to examine differences by ER status or morphology was low in our cohort because of the relative uncommonness of ER-negative and non-ductal-type tumours. Some studies have tended to demonstrate stronger risks for ER-positive breast cancer [12, 16, 20, 31], but none have shown significant interactions, and the literature is inconclusive [2]. We observed larger HRs for smoking and pre-menopausal, relative to post-menopausal, breast cancer, but the former did not reach statistical significance, and although the literature is variable, it does in general suggest a greater relative risk among pre-menopausal women [1, 2]. However, we found no evidence for a significant interaction with menopausal status, similar to other studies [8, 11, 32].

### Confounding by alcohol

Alcohol consumption was associated with smoking and is itself a known risk factor for breast cancer [7]. We adjusted for alcohol intake, and although this reduced the strength of the association between smoking and breast cancer (from HR 1.17 to 1.14), the association remained raised and significant. There is, however, concern that statistical adjustment using self-reported alcohol consumption may not be adequate to control fully for confounding by alcohol [7], so to explore further the extent of potential confounding, we stratified by alcohol consumption (Table 4). Within each stratum of consumption (< 20 g/day, 20–40 g/day, and 40 to < 60 g/day), the difference in self-reported alcohol intake between never and ever smokers was ≈ 1 g/day, and we calculated that this difference in consumption would be associated with a < 1% change in relative risk of breast cancer (using the alcohol-breast cancer estimate of relative risk from a large collaborative re-analysis [7]). Within each of these strata, it would require ever smokers to be drinking 20 g/day more than never smokers to produce a difference of ≈ 15% (similar to the 12–17% we saw). This implies that the association we observed between ever smoking and breast cancer may be too large to be explained by differences in alcohol intake alone.

We saw no significant association between smoking and breast cancer risk among non-drinkers, in concordance with a collaborative re-analysis of 43 case-control and 10 cohort studies [7], the American Cancer Society’s Cancer Prevention Study II cohort [16], and a subsequent pooled analysis of 14 cohort studies [8]. It is possible that there may be synergistic interaction between ever smoking and alcohol consumption, and risk of breast cancer, although only one study has shown the interaction as statistically significant [8]. There is some precedent to invoke synergism between smoking and alcohol because, for example, there is an established positive interaction between these two exposures and the aetiology of head and neck cancers [33]. However, non-drinking may occur for cultural or religious reasons, or because of underlying illness or other health issues, and in the United Kingdom at least, non-drinkers are a minority group; therefore, this potential interaction could be a reflection of a particular distribution of breast cancer risk factors among non-drinkers (and inadequate control for confounding among drinkers). Conversely, three other cohort studies demonstrated significantly raised risk among non-drinkers [18, 26, 29], although in two the increased raised risks were only in subgroups [26, 29].

### Smoking in adolescence

On the basis of epidemiological considerations and animal studies, the period from puberty to first birth may represent a window of particular susceptibility to breast cancer [34–37]. At puberty, the breast is made up of mainly undifferentiated terminal ductal and lobular structures which animal studies show are sensitive to chemical carcinogenesis [34]. At these young ages, ionizing radiation exposure also increases risk of breast cancer [37], especially if exposure is within 6 months of menarche [38]. We found that risk of breast cancer in ever smokers was greatest if smoking started at age < 17 years or started at peri-menarcheal or, more weakly, at peri-thelarcheal ages. A number of other studies have also demonstrated raised risks if smoking started in adolescence [8–13, 16–18, 20, 26, 28, 29, 32] or around menarche [11, 16, 26]. However, when we adjusted for pack-years of smoking, the raised risks for starting smoking close to age at menarche or thelarche were somewhat attenuated, suggesting over-adjustment (because of possible correlation between age starting smoking and pack-years) or confounding by pack-years. Previous studies have not made this adjustment, so the relative importance of early initiation or pack-years of use remains unclear.

### Smoking before first childbirth

Young age at first birth and increasing parity confer long-term protection against breast cancer [34, 35], and animal models point to terminal differentiation of breast tissue at full-term pregnancy being important in this process [34–36]. Increased risks have been reported for invasive breast cancer if smoking started before first childbirth [8–11, 16, 17, 20, 26, 28, 29, 32], but we found the association was significant only if we did not adjust for age at first pregnancy. Researchers in a number of previous studies have adjusted for age at first pregnancy and still found significant associations with interval to first birth [8, 9, 11–13, 16–18, 20, 26, 28, 29]; however, it is difficult to determine the adequacy of adjustment. For example, in a large pooled analysis of 14 cohort studies, there was a strong trend with smoking interval before first birth after adjustment for potential confounders that included age at first birth and number of live births (*P* = 0.0000002), whereas after stratification by age at first birth, the trends in each stratum were weaker (*P* = 0.12, 0.02, and 0.28) [8], which is suggestive of confounding.

### Interaction with family history

We found the association between smoking and breast cancer was significantly larger among women with a family history of the disease than in those without. Investigators in five previous studies have reported on this interaction with family history. Two studies showed no significant interaction but the researchers did not present stratified results, so we cannot determine if the direction of interaction supports or contradicts our findings [16, 19]. In three studies, researchers reported significant interactions, with one showing increased breast cancer risk with smoking only among those with a positive family history [39], whereas two demonstrated that breast cancer risk was raised only among those with no family history [15, 18]. Increased risk of breast cancer with smoking has also been seen in some [40, 41], but not all (*see* review [1] and a large meta-analysis [41]), studies of *BRCA1/2* carriers (or by proxy, women with three or more first-degree relatives with breast or ovarian cancer [42]). There are also reports of significant interactions with smoking and polymorphisms in carcinogen metabolism genes *NAT2* [43] and *CYP1A1* [44, 45] as well as breast cancer susceptibility single-nucleotide polymorphisms [46, 47]. Moreover, BRCA1 and BRCA2 proteins are involved in the repair of DNA damage, and it is therefore possible that *BRCA1/2* carriers may be more sensitive to effects of carcinogens in cigarette smoke. Thus, despite the limited and inconsistent literature, it is possible that there are gene-smoking interactions in relation to breast cancer risk (as there is, for instance, with bladder cancer [48]), and studies may benefit from being focussed on more detailed measures and timing of exposure (e.g., peri-menarcheal smoking or pack-years of use) rather than just ever/never smoking.

As in previous studies, we excluded from analysis women with prevalent breast or other malignant cancer [11–13, 15–17, 20, 28, 32] or prevalent in situ breast cancer [13] at recruitment; we restricted the analysis to invasive breast cancer [7–18, 20, 26, 28, 30]; and we adjusted for menopausal status and BMI [8, 10, 11, 13, 16, 18–20, 26, 30, 31], potential confounding variables that may also be influenced by smoking. There was little scope for bias from unascertained mortality or exits, or for erroneous reporting of breast cancer, because follow-up for vital and breast cancer status was obtained for 99% of participants and confirmation of reported breast cancers for over 99%. Our smoking information was gained at recruitment and from follow-up questionnaires 6 years later, and we were able to update smoking status, so that women who gave up smoking were classified as former smokers from that point in time. Only a small number of other cohort studies [13, 16, 20] have been able to update smoking exposure through follow-up. One limitation of our study is that we have no direct information on passive (secondhand) smoking, and therefore our risk estimates might be underestimated if never smokers were exposed to passive smoking and if this exposure affects risk of breast cancer [49].

If our results are not due to chance, residual confounding, or unidentified bias, they suggest that certain biological mechanisms deserve further attention, such as those involving exposure at peri-menarcheal ages, and gene-environment interactions, either of which may be the direct result of chemical carcinogenesis or an indirect consequence on hormonal pathways during this susceptible period of breast development.

## Conclusions

We found that smoking was associated with a modest but significantly increased risk of breast cancer, particularly among those who started at adolescent or peri-menarcheal ages, and that the relative risk of breast cancer associated with smoking was significantly greater for women with a family history of the disease.

## Additional file

Additional file 1: Table S1. Further characteristics of 102,927 women from the Generations Study who were recruited 2003–2013. **Table S2.** Characteristics of incident invasive breast cancer cases arising in 102,927 women from the Generations Study. **Table S3.** Relative risk of breast cancer in relation to smoking, by morphological type. **Table S4.** Relative risk of breast cancer in relation to former smoking, by level of alcohol consumption. **Table S5.** Relative risk of breast cancer in relation to smoking, by parity. **Table S6.** Relative risk of breast cancer in relation to smoking, by menopausal status. (DOCX 72 kb)

## Abbreviations

BMI: Body mass index
ER: Oestrogen receptor
MHT: Menopausal hormone therapy
OC: Oral contraceptive
