# Supplementary material for: Smoking and risk of breast cancer in the Generations Study cohort
Source: Breast Cancer Res. 2017 Nov 22;19:118. doi: 10.1186/s13058-017-0908-4 (PMC5698948; doi:10.1186/s13058-017-0908-4)
Supplement: Additional file 1: Table S1. — Further characteristics of 102,927 women from the Generations Study who were recruited 2003–2013. Table S2. Characteristics of incident invasive breast cancer cases arising in 102,927 women from the Generations Study. Table S3. Relative risk of breast cancer in relation to smoking, by morphological type. Table S4. Relative risk of breast cancer in relation to former smoking, by level of alcohol consumption. Table S5. Relative risk of breast cancer in relation to smoking, by parity. Table S6. Relative risk of breast cancer in relation to smoking, by menopausal status. (DOCX 72 kb) [file 13058_2017_908_MOESM1_ESM.docx]

| **TABLE S1**: Further characteristics of 102,927 women from the Generations Study who were recruited 2003–2013 | | | |
| --- | --- | --- | --- |
| **Study population** |  | **N** | ***%*** |
| Age started smoking (years) | | | |
| Never smoker |  | 66013 | *64.1* |
| <16 |  | 13773 | *13.4* |
| 17–19 |  | 14351 | *13.9* |
| 20+ |  | 7306 | *7.1* |
| Smoker, age started unknown |  | 1484 | *1.4* |
| Age at thelarche (years) | | | |
| 7–11 |  | 33251 | *32.3* |
| 12–14 |  | 48089 | *46.7* |
| 15–19 |  | 3440 | *3.3* |
| Not known |  | 18147 | *17.6* |
| Parity, at entry to cohort | | | |
| Nulliparous |  | 29209 | *28.4* |
| Parous |  | 73718 | *71.6* |
| *Age at 1^st^ parous birth (years)* |  |  |  |
| 13–19 |  | 4170 | *5.7* |
| 20–24 |  | 20520 | *27.8* |
| 25–29 |  | 30108 | *40.8* |
| 30–34 |  | 14721 | *20.0* |
| 35–49 |  | 4199 | *5.7* |
| Menopausal status at entry to cohort | | | |
| Pre-menopausal |  | 59841 | *58.1* |
| Post-menopausal |  | 43086 | *41.9* |
| BMI at age 20 | | | |
| <20 |  | 22808 | *22.2* |
| 20–<25 |  | 51217 | *49.8* |
| 25–<30 |  | 7463 | *7.3* |
| 30–<35 |  | 1307 | *1.3* |
| 35+ |  | 396 | *0.4* |
| Unknown |  | 19746 | *19.2* |
| Post-menopausal BMI at entry to cohort (*N*=43,086) | | | |
| <20 |  | 1671 | *3.9* |
| 20–<25 |  | 19064 | *44.3* |
| 25–<30 |  | 14856 | *34.5* |
| 30–<35 |  | 4710 | *10.9* |
| 35+ |  | 1741 | *4.0* |
| Unknown |  | 1044 | *2.4* |
| Total number of subjects |  | 102927 | *100.0* |

| **TABLE S2**: Characteristics of incident invasive breast cancer cases arising in 102,927 women from the Generations Study | | | |
| --- | --- | --- | --- |
| **Study population** |  | **N** | ***%*** |
| Age at breast cancer diagnosis (years) | | | |
| 24–44 |  | 247 | *13.6* |
| 45–54 |  | 474 | *26.1* |
| 55–64 |  | 610 | *33.6* |
| 65–74 |  | 404 | *22.3* |
| 75–95 |  | 80 | *4.4* |
| Year of breast cancer diagnosis | | | |
| 2004–2006 |  | 130 | *7.2* |
| 2007–2009 |  | 550 | *30.3* |
| 2010–2012 |  | 697 | *38.4* |
| 2013–2015 |  | 438 | *24.1* |
| Confirmation of breast cancer | | | |
| Confirmed* |  | 1813 | *99.9* |
| Self-reported only† |  | 2 | *0.1* |
| Morphological type | | | |
| Ductal |  | 1430 | *78.8* |
| Lobular |  | 298 | *16.4* |
| Tubular |  | 31 | *1.7* |
| Mucinous or colloid |  | 18 | *1.0* |
| Adenocarcinoma (not otherwise specified) |  | 15 | *0.8* |
| Other known types |  | 16 | *0.9* |
| Missing |  | 7 | *0.4* |
| Estrogen receptor status | | | |
| Positive |  | 1509 | *82.7* |
| Negative |  | 294 | *16.1* |
| Not tested |  | 6 | *0.3* |
| Missing |  | 6 | *0.3* |
| Total number of cases |  | 1815 | *100.0* |
| * Confirmation through national cancer registration or medical records  † With reported treatments that imply self-reported diagnosis of breast cancer is correct | | | |

| **TABLE S3**: Relative risk of breast cancer in relation to smoking, by morphological type | | | | | | | | | | | | | | |
| --- | --- | --- | --- | --- | --- | --- | --- | --- | --- | --- | --- | --- | --- | --- |
|  | **Morphological type*** | | | | | | | | | | | | | |
|  | **Ductal** | | | |  | **Lobular** | | | |  | **Other types, and missing** | | | |
|  | **Cases** | **HR**† | **95% CI** | ***P*-value** |  | **Cases** | **HR**† | **95% CI** | ***P*-value** |  | **Cases** | **HR**† | **95% CI** | ***P*-value** |
| **Ever previously smoked cigarettes**‡ | | | | | | | | | | | | | | |
| Never | 849 | 1.00 | Baseline |  |  | 174 | 1.00 | Baseline |  |  | 50 | 1.00 | Baseline |  |
| Ever | 581 | 1.12 | 1.01–1.25 | 0.033 |  | 124 | 1.16 | 0.92–1.47 | 0.20 |  | 37 | 1.24 | 0.81–1.90 | 0.33 |
|  | Ever smoking­ by morphological type interaction *P*=0.63 | | | | | | | | | | | | | |
| **Cigarettes per day, averaged over years when smoking** § | | | | | | | | | | | | | | |
| Never smoked | 849 | 1.00 | Baseline |  |  | 174 | 1.00 | Baseline |  |  | 50 | 1.00 | Baseline |  |
| 1–4 | 204 | 1.08 | 0.93–1.26 | 0.34 |  | 40 | 1.01 | 0.71–1.42 | 0.98 |  | 14 | 1.29 | 0.71–2.34 | 0.40 |
| 5–9 | 107 | 1.22 | 0.99–1.50 | 0.058 |  | 17 | 0.96 | 0.58–1.58 | 0.88 |  | 11 | 2.17 | 1.13–4.16 | 0.020 |
| 10–14 | 58 | 1.21 | 0.93–1.59 | 0.16 |  | 22 | 2.36 | 1.51–3.68 | 0.0001 |  | 2 | 0.71 | 0.17–2.93 | 0.64 |
| 15+ | 52 | 1.56 | 1.17–2.07 | 0.0023 |  | 9 | 1.39 | 0.71–2.71 | 0.34 |  | 1 | 0.51 | 0.07–3.66 | 0.50 |
| Unknown | 160 | 1.02 | 0.86–1.21 | 0.84 |  | 36 | 1.09 | 0.76–1.57 | 0.64 |  | 9 | 0.99 | 0.49–2.02 | 0.98 |
|  | Trend ǁ *P*=0.034 | | | |  | Trend ǁ *P*=0.0090 | | | |  | Trend ǁ *P*=0.27 | | | |
|  | Trend interaction *P*=0.075 | | | | | | | | | | | | | |
| **Duration of smoking (years)** ¶ | | | | | | | | | | | | | | |
| Never smoked | 849 | 1.00 | Baseline |  |  | 174 | 1.00 | Baseline |  |  | 50 | 1.00 | Baseline |  |
| 1–9 | 145 | 1.03 | 0.87–1.23 | 0.72 |  | 21 | 0.75 | 0.48–1.18 | 0.22 |  | 11 | 1.35 | 0.70–2.63 | 0.37 |
| 10–19 | 176 | 1.19 | 1.01–1.41 | 0.039 |  | 36 | 1.24 | 0.87–1.78 | 0.24 |  | 13 | 1.52 | 0.83–2.80 | 0.18 |
| 20–29 | 105 | 1.14 | 0.93–1.41 | 0.21 |  | 30 | 1.58 | 1.07–2.34 | 0.022 |  | 6 | 1.13 | 0.48–2.65 | 0.77 |
| 30+ | 126 | 1.24 | 1.03–1.50 | 0.026 |  | 28 | 1.20 | 0.80–1.80 | 0.37 |  | 5 | 0.84 | 0.34–2.12 | 0.72 |
| Duration unknown | 29 | 0.86 | 0.59–1.24 | 0.42 |  | 9 | 1.25 | 0.64–2.45 | 0.52 |  | 2 | 1.03 | 0.25–4.27 | 0.96 |
|  | Trend§ *P*=0.35 | | | |  | Trend§ *P*=0.15 | | | |  | Trend § *P*=0.55 | | | |
|  | Trend interaction *P*=0.48 | | | | | | | | | | | | | |
| **Pack-years of smoking** § | | | | | | | | | | | | | | |
| Never smoked | 849 | 1.00 | Baseline |  |  | 174 | 1.00 | Baseline |  |  | 50 | 1.00 | Baseline |  |
| 1–<5 | 149 | 1.13 | 0.95–1.34 | 0.18 |  | 24 | 0.91 | 0.60–1.40 | 0.68 |  | 9 | 1.18 | 0.58–2.41 | 0.65 |
| 5–<10 | 77 | 0.99 | 0.78–1.25 | 0.90 |  | 16 | 1.02 | 0.61–1.71 | 0.93 |  | 10 | 2.20 | 1.12–4.35 | 0.023 |
| 10–<20 | 104 | 1.21 | 0.98–1.49 | 0.078 |  | 26 | 1.47 | 0.97–2.22 | 0.069 |  | 8 | 1.60 | 0.76–3.38 | 0.22 |
| 20+ | 91 | 1.49 | 1.19–1.85 | 0.0004 |  | 22 | 1.61 | 1.03–2.51 | 0.035 |  | 1 | 0.28 | 0.04–2.06 | 0.21 |
| Unknown | 160 | 1.02 | 0.86–1.21 | 0.83 |  | 36 | 1.09 | 0.76–1.57 | 0.63 |  | 9 | 0.99 | 0.49–2.01 | 0.98 |
|  | Trend§ *P*=0.0221 | | | |  | Trend§ *P*=0.026 | | | |  | Trend ǁ *P*=0.12 | | | |
|  | Trend interaction *P*=0.063 | | | | | | | | | | | | | |
| **Current or former smoking** ‡ | | | | | | | | | | | | | | |
| Never smoked | 849 | 1.00 | Baseline |  |  | 174 | 1.00 | Baseline |  |  | 50 | 1.00 | Baseline |  |
| Current** | 68 | 1.09 | 0.85–1.40 | 0.512 |  | 15 | 1.28 | 0.75–2.17 | 0.36 |  | 4 | 1.06 | 0.38–2.98 | 0.91 |
| Former | 513 | 1.13 | 1.01–1.26 | 0.033 |  | 109 | 1.15 | 0.90–1.46 | 0.26 |  | 33 | 1.26 | 0.81–1.97 | 0.31 |
|  | Smoking­ status by morphological type interaction *P*=0.97 | | | | | | | | | | | | | |
| **Time since cessation (years)** ¶ | | | | | | | | | | | | | | |
| Never smoked | 849 | 1.00 | Baseline |  |  | 174 | 1.00 | Baseline |  |  | 50 | 1.00 | Baseline |  |
| Current** smoker | 68 | 1.09 | 0.85–1.40 | 0.49 |  | 15 | 1.30 | 0.76–2.20 | 0.34 |  | 4 | 1.07 | 0.38–2.99 | 0.90 |
| 1–9 | 102 | 1.27 | 1.03–1.56 | 0.026 |  | 17 | 1.23 | 0.75–2.02 | 0.42 |  | 8 | 1.70 | 0.82–3.49 | 0.15 |
| 10–19 | 105 | 1.09 | 0.89–1.34 | 0.40 |  | 33 | 1.85 | 1.27–2.70 | 0.0013 |  | 7 | 1.24 | 0.56–2.73 | 0.60 |
| 20–29 | 126 | 1.12 | 0.93–1.36 | 0.23 |  | 21 | 0.87 | 0.55–1.36 | 0.53 |  | 6 | 0.88 | 0.37–2.08 | 0.77 |
| 30+ | 165 | 1.12 | 0.94–1.33 | 0.20 |  | 32 | 0.90 | 0.62–1.32 | 0.60 |  | 12 | 1.52 | 0.78–2.96 | 0.22 |
| Duration unknown | 15 | 0.84 | 0.51–1.40 | 0.51 |  | 6 | 1.59 | 0.70–3.61 | 0.26 |  | 0 |  |  |  |
|  | Trend§ P=0.26 | | | |  | Trend§ P=0.039 | | | |  | Trend § *P*=0.90 | | | |
|  | Trend heterogeneity *P*=0.38 | | | | | | | | | | | | | |
| **Age started smoking among ever smokers (years)** | | | | | | | | | | | | | | |
| Never smoked | 849 | 1.00 | Baseline |  |  | 174 | 1.00 | Baseline |  |  | 50 | 1.00 | Baseline |  |
| <17 | 205 | 1.22 | 1.05–1.43 | 0.012 |  | 38 | 1.17 | 0.82–1.66 | 0.38 |  | 18 | 1.83 | 1.07–3.14 | 0.027 |
| 17–19 | 239 | 1.15 | 0.99–1.33 | 0.062 |  | 54 | 1.24 | 0.91–1.69 | 0.17 |  | 11 | 0.92 | 0.48–1.76 | 0.81 |
| 20+ | 116 | 0.98 | 0.80–1.19 | 0.81 |  | 29 | 1.12 | 0.75–1.67 | 0.57 |  | 6 | 0.87 | 0.37–2.03 | 0.74 |
| Age unknown | 21 | 0.97 | 0.63–1.49 | 0.88 |  | 3 | 0.64 | 0.20–2.01 | 0.44 |  | 2 | 1.63 | 0.39–6.77 | 0.50 |
|  | Trend§ *P*=0.22 | | | |  | Trend§ *P*=0.74 | | | |  | Trend among ever smokers§ *P*=0.95 | | | |
|  | Trend interaction *P*=0.95 | | | | | | | | | | | | | |
| * Based the International Classification of Diseases for Oncology: Morphology of Neoplasms codes (Ductal: 850_/3 ; Lobular: 852_/3 except 8521/3 ; Other types, and missing includes 8 cases where morphology is missing)  † Adjusted for: attained age (Cox regression time scale); time since recruitment to cohort (0, 1–2, 3+ years); birth cohort (1908–39, 1940–49, 1950–59, 1960–69, 1970–96); benign breast disease (yes, no); family history of breast cancer in 1st degree relatives (yes, no); socio-economic score (ACORN score as trend, missing); age at menarche (trend, missing); age at first pregnancy (trend, missing); parity (trend, missing); duration of breastfeeding (trend, missing); current oral contraceptive use before menopause (yes, no); alcohol consumption (never regular, trend current drinker 1­– <60g/day, current drinker 60+g/day, past drinker, drinker with unknown details); physical activity (log(metabolic equivalent) trend, missing ); pre-menopausal body mass index at age 20 years (trend, missing); post-menopausal body mass index (trend, missing); menopausal hormone therapy use (never used, ex-user, current estrogen only user, current estrogen plus progestogen user, current user of other types, missing); menopausal status (pre- or post-menopausal) and age at menopause (trend, missing).  ‡ Time updated through follow-up period  § Time updated at the point renewed information was available from follow-up questionnaire  ǁ Trend excludes Never smoked and Unknown group  ¶ Time updated in yearly steps  ** Includes current smokers and <1 year immediately after stopping | | | | | | | | | | | | | | |

| **TABLE S4:** Relative risk of breast cancer in relation to former-smoking, by level of alcohol consumption | | | | | |
| --- | --- | --- | --- | --- | --- |
| **Strata** |  | **Total number of cases in strata** | **HR for former-smoking relative to never smoking within strata of alcohol consumption*** | **95% CI** | ***P*-value** |
| **Alcohol**† | | | | | |
| Non-drinker |  | 103 | 0.83 | 0.49–1.39 | 0.48 |
| Ever drinker |  | 1625 | 1.18 | 1.07–1.31 | 0.0010 |
|  |  |  | Interaction *P* ‡=0.19 | | |
| **Alcohol**† | | | | | |
| Non-drinker |  | 103 | 0.83 | 0.49–1.39 | 0.47 |
| <20g/day |  | 685 | 1.18 | 1.01–1.38 | 0.041 |
| 20­–<40g/day |  | 339 | 1.20 | 0.97–1.49 | 0.091 |
| 4­0–<60g/day |  | 77 | 1.07 | 0.68–1.67 | 0.78 |
| ≥60g/day |  | 24 | 2.04 | 0.81–5.15 | 0.13 |
| Current, amount unknown |  | 170 | 1.49 | 1.10–2.04 | 0.011 |
| Former drinker |  | 278 | 0.96 | 0.75–1.24 | 0.76 |
| Drinker, details missing |  | 52 | 0.72 | 0.35–1.48 | 0.37 |
|  |  |  | Interaction *P* ‡=0.21 | | |
| * Adjusted for: attained age (Cox regression time scale); time since recruitment to cohort (0, 1–2, 3+ years); birth cohort (1908–39, 1940–49, 1950–59, 1960–69, 1970–96); benign breast disease (yes, no); family history of breast cancer in 1st degree relatives (yes, no); socio-economic score (ACORN score as trend, missing); age at menarche (trend, missing); age at first pregnancy (trend, missing); parity (trend, missing); duration of breastfeeding (trend, missing); current oral contraceptive use before menopause (yes, no); physical activity (log(metabolic equivalent) trend, missing ); pre-menopausal body mass index at age 20 years (trend, missing); post-menopausal body mass index (trend, missing); menopausal hormone therapy use (never used, ex-user, current estrogen only user, current estrogen plus progestogen user, current user of other types, missing); menopausal status (pre- or post-menopausal) and age at menopause (trend, missing).  † Time updated through follow-up period  ‡ Interaction across all categories, including missing groups | | | | | |

| **TABLE S5**: Relative risk of breast cancer in relation to smoking, by parity* | | | | | | | | | |
| --- | --- | --- | --- | --- | --- | --- | --- | --- | --- |
|  | **Parity** | | | | | | | | |
|  | **Nulliparous** | | | |  | **Parous** | | | |
|  | **Cases** | **HR†** | **95% CI** | ***P*-value** |  | **Cases** | **HR†** | **95% CI** | ***P*-value** |
| **Ever previously smoked cigarettes*** | | | | | | | | | |
| Never | 146 | 1.00 | Baseline |  |  | 927 | 1.00 | Baseline |  |
| Ever | 119 | 1.37 | 1.07–1.75 | 0.012 |  | 623 | 1.09 | 0.99–1.21 | 0.089 |
|  | Ever smoking­ by parity interaction *P*=0.095 | | | | | | | | |
| **Age started smoking** * | | | | | | | | | |
| Never smoked | 146 | 1.00 | Baseline |  |  | 927 | 1.00 | Baseline |  |
| <20 | 90 | 1.51 | 1.16–1.97 | 0.0022 |  | 475 | 1.13 | 1.01–1.27 | 0.033 |
| 20+ | 28 | 1.15 | 0.77–1.73 | 0.49 |  | 123 | 0.99 | 0.82–1.20 | 0.91 |
| Age unknown | 1 | 0.32 | 0.04–2.28 | 0.25 |  | 25 | 1.01 | 0.68–1.51 | 0.95 |
|  | Age started smoking­ by parity interaction *P*=0.10 | | | | | | | | |
| **Starting smoking relative to age at menarche (years)** | | | | | | | | | |
| Never smoked | 146 | 1.00 | Baseline |  |  | 927 | 1.00 | Baseline |  |
| Before or <5 years | 48 | 1.64 | 1.18–2.28 | 0.0032 |  | 252 | 1.16 | 1.00–1.34 | 0.050 |
| 5+ years | 58 | 1.27 | 0.94–1.73 | 0.12 |  | 298 | 1.08 | 0.94–1.24 | 0.26 |
| Interval unknown | 13 | 1.07 | 0.60–1.91 | 0.82 |  | 73 | 0.97 | 0.75–1.27 | 0.84 |
|  | Age started smoking­ relative to menarche by parity interaction *P*=0.10 | | | | | | | | |
|  |  | | | | | | | | |
| * Time updated through follow-up  † Adjusted for: attained age (Cox regression time scale); time since recruitment to cohort (0, 1–2, 3+ years); birth cohort (1908–39, 1940–49, 1950–59, 1960–69, 1970–96); benign breast disease (yes, no); family history of breast cancer in 1st degree relatives (yes, no); socio-economic score (ACORN score as trend, missing); age at menarche (trend, missing); current oral contraceptive use before menopause (yes, no); alcohol consumption (never regular, trend current drinker 1­– <60g/day, current drinker 60+g/day, past drinker, drinker with unknown details); physical activity (log(metabolic equivalent) trend, missing ); pre-menopausal body mass index at age 20 years (trend, missing); post-menopausal body mass index (trend, missing); menopausal hormone therapy use (never used, ex-user, current estrogen only user, current estrogen plus progestogen user, current user of other types, missing); menopausal status (pre- or post-menopausal) and age at menopause (trend, missing). | | | | | | | | | |

| **TABLE S6**: Relative risk of breast cancer in relation to smoking, by menopausal status* | | | | | | | | | |
| --- | --- | --- | --- | --- | --- | --- | --- | --- | --- |
|  | **Menopausal status** | | | | | | | | |
|  | **Premenopausal** | | | |  | **Postmenopausal** | | | |
|  | **Cases** | **HR**† | **95% CI** | ***P*-value** |  | **Cases** | **HR**† | **95% CI** | ***P*-value** |
| **Ever previously smoked cigarettes*** | | | | | | | | | |
| Never | 323 | 1.00 | Baseline |  |  | 750 | 1.00 | Baseline |  |
| Ever | 198 | 1.17 | 0.98–1.40 | 0.088 |  | 544 | 1.13 | 1.01–1.26 | 0.040 |
|  | Ever smoking­ by menopausal status interaction *P*=0.73 | | | | | | | | |
| **Age started smoking*** | | | | | | | | | |
| Never smoked | 323 | 1.00 | Baseline |  |  | 750 | 1.00 | Baseline |  |
| <20 | 162 | 1.21 | 1.00–1.46 | 0.051 |  | 403 | 1.19 | 1.05–1.34 | 0.0069 |
| 20+ | 28 | 0.93 | 0.63–1.36 | 0.70 |  | 123 | 1.02 | 0.84–1.23 | 0.86 |
| Age unknown | 8 | 1.56 | 0.77–3.14 | 0.22 |  | 18 | 0.80 | 0.50–1.28 | 0.35 |
|  | Age started smoking­ by menopausal status interaction *P*=0.49 | | | | | | | | |
| **Starting smoking relative to age at menarche (years)** | | | | | | | | | |
| Never smoked | 323 | 1.00 | Baseline |  |  | 750 | 1.00 | Baseline |  |
| Before or <5 years | 102 | 1.36 | 1.08–1.70 | 0.0080 |  | 198 | 1.19 | 1.01–1.40 | 0.035 |
| 5+ years | 76 | 1.07 | 0.83–1.37 | 0.61 |  | 280 | 1.11 | 0.96–1.28 | 0.15 |
| Interval unknown | 20 | 0.87 | 0.55–1.40 | 0.58 |  | 66 | 1.03 | 0.78–1.35 | 0.86 |
|  | Age started smoking­ relative to menarche by menopausal status interaction *P*=0.66 | | | | | | | | |
|  |  | | | | | | | | |
| * Time updated through follow-up  † Adjusted for: attained age (Cox regression time scale); time since recruitment to cohort (0, 1–2, 3+ years); birth cohort (1908–39, 1940–49, 1950–59, 1960–69, 1970–96); benign breast disease (yes, no); family history of breast cancer in 1st degree relatives (yes, no); socio-economic score (ACORN score as trend, missing); age at menarche (trend, missing); age at first pregnancy (trend, missing); parity (trend, missing); duration of breastfeeding (trend, missing); current oral contraceptive use before menopause (yes, no); alcohol consumption (never regular, trend current drinker 1­– <60g/day, current drinker 60+g/day, past drinker, drinker with unknown details); physical activity (log(metabolic equivalent) trend, missing ); pre-menopausal body mass index at age 20 years (trend, missing); post-menopausal body mass index (trend, missing); menopausal hormone therapy use (never used, ex-user, current estrogen only user, current estrogen plus progestogen user, current user of other types, missing). | | | | | | | | | |
